# A Study of Methylcellulose Based Polymer Electrolyte Impregnated with Potassium Ion Conducting Carrier: Impedance, EEC Modeling, FTIR, Dielectric, and Device Characteristics

**DOI:** 10.3390/ma14174859

**Published:** 2021-08-26

**Authors:** Muaffaq M. Nofal, Jihad M. Hadi, Shujahadeen B. Aziz, Mohamad A. Brza, Ahmad S. F. M. Asnawi, Elham M. A. Dannoun, Aziz M. Abdullah, Mohd F. Z. Kadir

**Affiliations:** 1 Department of Mathematics and General Sciences, Prince Sultan University, P.O. Box 66833, Riyadh 11586, Saudi Arabia; muaffaqnofal69@gmail.com; 2 Department of Medical Laboratory of Science, College of Health Sciences, University of Human Development, Sulaimaniyah 46001, Iraq; jihad.chemist@gmail.com; 3 Hameed Majid Advanced Polymeric Materials Research Laboratory, Physics Department, College of Science, University of Sulaimani, Qlyasan Street, Sulaimaniyah 46001, Iraq; mohamad.brza@gmail.com; 4 Department of Civil Engineering, College of Engineering, Komar University of Science and Technology, Sulaimaniyah 46001, Iraq; 5 Chemical Engineering Section, Malaysian Institute of Chemical & Bioengineering Technology (UniKL MICET), University Kuala Lumpur, Alor Gajah 78000, Malaysia; asyafiq.asnawi@s.unikl.edu.my; 6 General Science Department, Woman Campus, Prince Sultan University, P.O. Box 66833, Riyadh 11586, Saudi Arabia; elhamdannoun1977@gmail.com; 7 Department of Applied Physics, College of Medical and Applied Sciences, Charmo University, Peshawa Street, Chamchamal 46023, Iraq; aziz.abdullah@charmouniversity.org; 8 Centre for Foundation Studies in Science, University of Malaya, Kuala Lumpur 50603, Malaysia; mfzkadir@um.edu.my

**Keywords:** MC polymer electrolyte, impedance study, ion transport, ftir analysis, TNM, LSV, CV analyses

## Abstract

In this research, a biopolymer-based electrolyte system involving methylcellulose (MC) as a host polymeric material and potassium iodide (KI) salt as the ionic source was prepared by solution cast technique. The electrolyte with the highest conductivity was used for device application of electrochemical double-layer capacitor (EDLC) with high specific capacitance. The electrical, structural, and electrochemical characteristics of the electrolyte systems were investigated using various techniques. According to electrochemical impedance spectroscopy (EIS), the bulk resistance (*R_b_*) decreased from 3.3 × 10^5^ to 8 × 10^2^ Ω with the increase of salt concentration from 10 wt % to 40 wt % and the ionic conductivity was found to be 1.93 ×10^−5^ S/cm. The dielectric analysis further verified the conductivity trends. Low-frequency regions showed high dielectric constant, *ε*′ and loss, *ε*″ values. The polymer-salt complexation between (MC) and (KI) was shown through a Fourier transformed infrared spectroscopy (FTIR) studies. The analysis of transference number measurement (TNM) supported ions were predominantly responsible for the transport process in the MC-KI electrolyte. The highest conducting sample was observed to be electrochemically constant as the potential was swept linearly up to 1.8 V using linear sweep voltammetry (LSV). The cyclic voltammetry (CV) profile reveals the absence of a redox peak, indicating the presence of a charge double-layer between the surface of activated carbon electrodes and electrolytes. The maximum specific capacitance, *C_s_* value was obtained as 118.4 F/g at the sweep rate of 10 mV/s.

## 1. Introduction

Besides improving energy and power efficiency, one of the remaining challenges in the development of energy storage systems, including smart grids, portable electronic devices, and hybrid vehicles, is to minimize manufacturing costs and reduce environmental pollution [1]. A more recent emphasis has been focused on solid polymer electrolytes (SPEs) as an alternative conventional organic sol–gel electrolyte. Dimensional stability, durability, a comparatively wide potential window above 1.5 V, and eco-friendliness are all properties of these materials [2]. In the technology area, natural polymers for fabricating SPEs have gained interest for application in electrochemical devices such as electrical double-layer capacitors (EDLCs) and proton batteries. Due to their exceptional chemical and mechanical performances, many studies have shown that natural SPEs exhibit a good potential for device applications [3,4]. Natural polymers are defined as materials that extensively happen in nature or are obtained from animals or plants. Natural polymers are vital to way of life as our human forms are based on them. Some of the examples of natural polymers are nucleic acid and proteins that happen in human body, natural rubber, silk, and methylcellulose (MC). MC is known to be competitively marketed and is environmentally safe. It has suitable film-forming characteristics with good mechanical and electrical properties. Through dative bonds, cations can interact with oxygen atoms of MC. As a consequence, MC comprises functional groups, such as alcohol (R-OH), ether (R-O-R), and ester (RCOOR) groups which are promising as an ion conduction mechanism due to their single pair of electrons. MC is also considered an amorphous polymer with its comparatively high glass transition temperature [5,6].

Supercapacitors consist of two porous electrodes separated by an ionically conducting electrolyte. The electrodes could be made of substances including polymers, carbon and metal oxides. Supercapacitors can be a favorable energy conversion device for a wide range of applications, where significant amounts of energy must be stored or released in a short period. A supercapacitor is classified into three major types, namely pseudo-capacitors, EDLCs, and hybrid capacitors. Pseudo-capacitors undergo a fast Faradaic mechanism [7], some examples of which include under potential deposition, intercalation, and reduction-oxidation reactions using metal oxide-based electrodes or electroactive conducting polymer. However, EDLCs do not involve any Faradaic mechanisms. EDLCs only require the accumulation of ions induced by the adsorption of charge carrier at the electrode/electrolyte interfaces. Owing to the storage process, EDLC is the non-Faradaic mechanism [8]. The main features of EDLCs, such as reliability, high energy capacity, reversibility, and safety improvements have drawn considerable interest, and making it a strong choice for various applications [9].

Activated carbon electrodes play a crucial role in the fabricating of EDLC due to their good chemical and physical properties such as low cost and easy availability, and high conductivity above 10^−4^ S/cm, which can be manufactured from a diversity of precursors [10,11]. As a result, coal is the most common supply of activated carbon production due to its availability, high content of carbons from 60% to 80%, and cost-effectiveness [12,13].

T.-Y. Chen et al. [14] electrodeposited NiSe nanoparticles on a carbon nanotube (CNT) forest to prepare a porous and intertwined network (denoted as CNT@NiSe/stainless steel (SS)). They then used the CNT@NiSe/SS as a free-standing and multifunctional electrode for supercapacitor (SC) application. The CNT@NiSe/SS composite electrode showed excellent capacity retention of 85%, and higher specific capacity of 126 mA h g^−1^ (1007 F g^−1^) in comparison with individual CNTs and NiSe. Lien et al. [15] developed a co-solvent-in-deep eutectic solvent (DES) system by mixing acetonitrile and water with a typical DES electrolyte composed of lithium perchlorate and acetamide. They have also used hydrogel composed of reduced graphene oxide (rGO) and 1T(trigonal)-MoS2 as the electrode materials for SC application. The authors fabricated high voltage symmetric supercapacitors using hydrogel and hybrid DES as the electrode and electrolyte materials, respectively. The SC at an operating voltage of 2.3 V achieved the maximum energy density of 31.2 Wh/kg at a power density of 1164 W/kg. The fabricated SC also showed 91% capacitance retention after 20,000 cycles. Hsiang et al. [16] presented rationally materials design of an optimum NiCo_2_S_4_ nanoparticle in a rGO matrix as a NiCo_2_S_4_/rGO nanocomposite. The authors reported the enhancements in the materials technology, showing the NiCo_2_S_4_/rGO nanocomposite electrode material with a very good specific capacitance of 963–700 F/g at 1–15 A/g, long cycle life of 3000 cycles, and high capacitance retention of 70%.

**Figure 1:**
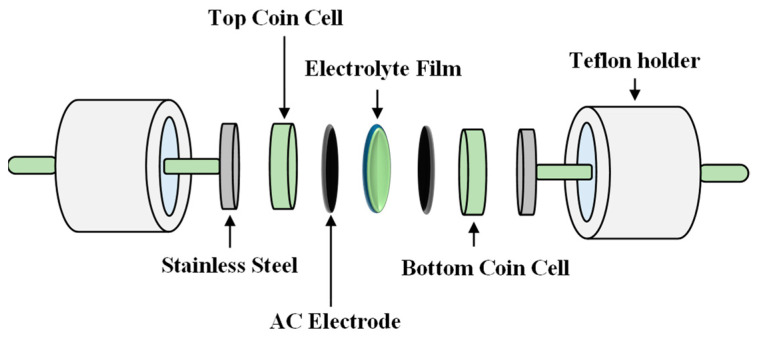
The schematic diagram of an EDLC cell. Adapted from reference [4].

Adding inorganic salt to a polymer provides ion mobility and the polymer host chain plays a crucial role in the ion transport mechanism of the polymer electrolytes. Consequently, ion motion arises across the amorphous area, which is aided by the segmental motion of the polymer chains [17]. The use of potassium complexed electrolyte films has been discovered to have some benefits over their lithium counterparts. Nadimicherla et al. [18] reported that the smaller ions such as (Li^+^ and Mg^2+^) possess lower mobility compared to the larger cations of (K^+^ and Zn^2+^) in polymer-based electrolytes. The smaller cations are entrenched or captured by the polymeric network. Furthermore, lithium–ion interactions with the polar polymer chains are stronger than potassium ions, and thus lithium–ion transport involves higher activation energy of 97.4 kJ mol^−1^ [19]. The aim of this study is to prepare an SPE film using a biopolymer of MC doped with various concentrations of potassium iodide (KI) as the ionic source for application in EDLC device. We have investigated the effect of different KI concentration has on the conductivity of MC. Also, the electrolyte with the highest conductivity was employed in the EDLC and its decomposition potential and specific capacitance were investigated. Figure 1 depicts the schematic diagram of an EDLC cell. As seen in Figure 1, the electrolyte is inserted between two activated carbon (AC) electrodes and then packed in coin cells of CR2032 to fabricate the EDLC. The prepared EDLC device was sandwiched in a Teflon case holder with two stainless steel electrodes to investigate the capacitive behavior of the device. While measuring the impedance data of the films, the arrangement of the cell was stainless steel electrolyte film stainless steel.

## 2. Experimental Details

### 2.1. Materials and Electrolyte Preparation

MC powder was used as a host polymeric raw material and KI salt was used as the ionic source. Both reagents were purchased from Sigma-Aldrich (Kuala Lumpur, Malaysia). The electrolytes were prepared using a solution casting technique by dissolving 1 g MC in 50 mL distilling water, with constant stirring, at room temperature for ~3 h. Subsequently, various amounts of KI salt were added to the MC solutions separately. The solutions were stirred continuously until a homogenous polymer–salt complex was obtained. The quantity of salt was varied from 10 to 40 weight percent (wt %) in steps of ten to obtain MC-KI electrolytes. The electrolyte samples were correspondingly specified as MCKI0, MCKI1, MCKI2, MCKI3, and MCK4 for MC incorporated with 0, 10, 20, 30, and 40 wt % of KI. The choice of KI concentrations is based on the ability of the MC to accommodate and dissolve the salt. Eventually, the solutions were cast on four individual categorized glass Petri dishes and left at room temperature to slowly evaporate the solvent. The films were further dried by transferring the prepared films to a desiccator.

### 2.2. Impedance Spectroscopy and FTIR Study

Electrical impedance spectroscopy (EIS) at the SPE was conducted using a Z HI-tester (Nagano, Japan) at a DC potential was 0.04 V, onto which an Ac voltage of peak-to-peak amplitude 10 mV was superimposed, over a frequency range of 5 MH and 50 HZ.

The inductance-capacitance-resistance (LCR) meter (Z HI-tester) was used to study the solid polymer electrolyte’s electrical impedance spectroscopy (EIS) in the frequency range of (50 Hz ≤ *f* ≤ 5 MHz). The DC potential was 0.04 V. An SPE film of geometric area of 2.01 cm^2^ was kept between two stainless-steel electrodes by applying a spring pressure which is used to press the electrolyte films. The stainless-steel electrode was used as the working, reference, and counter electrodes while the reference and counter electrodes were combined together. The EIS data were fitted with the electric equivalent circuit (EEC) model. The common electrical elements such as resistors and capacitors are used in this model. The EEC model is simple method and provides the entire picture of the system [5].

A spotlight 400 Perkin-Elmer spectrometer (Malvern Panalytical Ltd., Malvern, UK) was employed to perform the Fourier Transforms Infrared (FTIR) spectroscopy measurements. The transmitting range was performed between 940 and 4000 cm^−1^ with a resolution of 2 cm^−1^.

It is vital to use Equation (1) to measure the DC ionic conductivity (*σ_dc_*) of the MCKI samples based on the bulk resistance (*R_b_*) value [20,21]

(1) $$\sigma_{dc}=\left(\frac{1}{R_{b}}\right)\times \left(\frac{t}{A}\right)$$

where *t* and *A* denote the sample thickness and electrode area, respectively. The dielectric constant (*ε*′) and dielectric loss (*ε*″) are obtained using Equations (2) and (3) [20,21].

(2) $$\varepsilon \prime =\frac{Z\prime \prime }{\left({Z\prime }^{2}+{Z\prime \prime }^{2}\right)C_{o}\omega }$$

(3) $$\varepsilon \prime \prime =\frac{Z\prime }{\left({Z\prime }^{2}+{Z\prime \prime }^{2}\right)C_{o}\omega }$$

where, *ω* and *C_o_* denote the angular frequency and capacitance, which are given by (*ω* *=* 2*πf*) and *ε_o_A*/*t*, respectively, where *ε_o_* stands for the free space permittivity, *A* the electrode area and *t* the thickness of the film [22].

The real and imaginary (*M_i_* and *M_r_*) parts of complex electric modulus (M*) were calculated using Equations (4) and (5) [23,24].

(4) $$M\prime =\left[\frac{\varepsilon \prime }{\left({\varepsilon \prime }^{2}+{\varepsilon \prime \prime }^{2}\right)}\right]=Z\prime \prime C_{o}\omega$$

(5) $$M\prime \prime =\left[\frac{\varepsilon \prime \prime }{\left({\varepsilon \prime }^{2}+{\varepsilon \prime \prime }^{2}\right)}\right]=Z\prime C_{o}\omega$$

### 2.3. Study of Transference Number Measurement (TNM) and Linear Sweep Voltammetry (LSV)

In TNM, two types of ionic transport, *t_ion_* and electron transport *t_el_* for the most conducting sample (MCKI4) were studied. A DP3003 digital DC power supply (V & A instrument, Shanghai, China) was employed to polarize the cell against time at room temperature by applying a working voltage of 0.2 V. Linear sweep voltammetry (LSV) was used to determine the maximum potential window for the (MCKI4) film using a Digi-IVY DY2300 potentiostat (Neware, Shenzhen, China). The scan rate was fixed at 10 mV/s, and then the sample was sandwiched between two stainless steel electrodes with Teflon holders. Equations (6) and (7) were used to measure the transport ions (*t_ion_*) and transport electrons (*t_el_*) of the MCKI4 film, as the film was positioned between two stainless-steel electrodes [25].

(6) $$t_{ion}=\frac{I_{i}-I_{ss}}{I_{i}}$$

(7) $$t_{el}=1-t_{ion}$$

where *I_i_* refers to the initial current, containing ions and electrons and *I_ss_* stands for the current of the steady-state that contains only electrons.

### 2.4. EDLC Fabrication

Typically, the ingredients used to prepare electrodes include solvent and carbonaceous materials. In preparing the EDLC electrodes, 0.25 g of carbon black, 3.25 g of activated carbon, and 0.5 g of polyvinylidene fluoride (PVdF) were dry mixed in a planetary ball miller (XQM-0.4, Fujian, China) at 500 rpm for ~20 min. Then, all powders were dissolved and stirred continuously in 20 mL of N-methyl pyrrolidone until it became a dark black solution. In the next step, the black solution was covered by an aluminum foil using a doctor blade technique. Subsequently, an oven was used to dry the coated aluminum foil for a specific time at ~60 °C. To eliminate any excess moisture, the electrodes were placed in a silica gel desiccator. The relatively uppermost conducting sample was located between a pair of activated carbon electrodes and packaged in coin cells of CR2032. Eventually, in order to perform cyclic voltammetry (CV) of the assembled EDLC, the Digi-IVY DY2300 potentiostat has been employed at various scan rates of 10, 20, 50, and 100 mV/s and charged from 0 to 0.9 V. The specific capacitance, *C_s_* for the assembled EDLC has been determined using Equation (8) [25].

(8) $$C_{cv}=\int_{V_{i}}^{V_{f}}\frac{I\left(V\right)dV}{2mv\left(V_{f}-V_{i}\right)}$$

where *V_i_* is the initial potential (i.e., 0 V), and *V_f_* is the final potential (i.e., 0.9 V), *m* and *υ* are the mass of active material and the potential sweep rates (mV/s), respectively. *I*(*V*)*dV* denotes the area under a cyclic voltammetric trace.

## 3. Result and Discussion

### 3.1. Impedance Study

**Figure 2:**
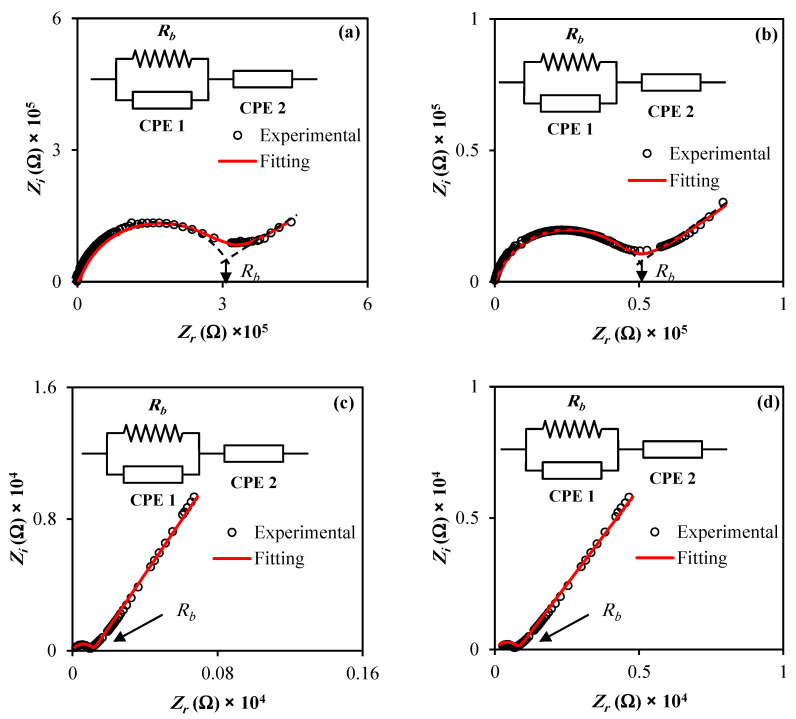
Impedance plots for (**a**) MCKI1, (**b**) MCKI2, (**c**) MCKI3, and (**d**) MCKI 4 electrolyte films.

Polymer electrolytes were commonly applied to devices as a part of an advanced material class. Impedance spectroscopy plays a crucial role in studying the electrical properties of a wide range of polymeric electrolyte materials. It is also a powerful technique for analyzing the ionic conductivity of new materials used in electrochemical energy systems, including EDLCs, charge transfer resistance, and diffusion layer. Plots of impedance spectra (*Z_i_* versus *Z_r_*) for the MCKI1, MCKI2, MCKI3, and MCKI4 systems are shown in Figure 2a–d. In general, the impedance responses are usually characterized by a semicircle in the high frequency region and a straight line in the low frequency region [26].

EIS data are commonly analyzed by fitting to an equivalent electrical circuit model (EEC). Most of the circuit elements in the model are common electrical elements such as resistors, capacitors, and inductors. To be useful, the elements in the model should have a basis in the physical electrochemistry of the system. The EEC method has been used to investigate the EIS because it is simple and shows the entire picture of the system [5,27]. The impedance diagrams in Figure 1 can generally be represented by an equivalent circuit consisting of a charge transfer resistance (*R_b_*) in a parallel arrangement with constant phase element 1 (CPE1) in high frequency region and in a series arrangement with constant phase element 2 (CPE2) in the low frequency region, as shown in the inset of Figure 1. The impedance arising from CPE, ZCPE, is expressed by Equation (9) [5,27]

(9) $$Z_{CPE}=\frac{1}{C\omega^{p}}\left[\cos\left(\frac{\pi p}{2}\right)-i\sin\left(\frac{\pi p}{2}\right)\right]$$

Here, *C* is the CPE capacitance, *ω* is the angular frequency and *p* is related to the EIS deviation from the imaginary axis. The *Z_r_* and *Z_i_* related to the EEC (insets of Figure 2a–d) are formulated by Equations (10) and (11)

(10) $$Z_{r}=\frac{R_{b}^{2}C_{1}\omega^{p_{1}}cos(\pi p_{1}/2)+R_{b}}{2R_{b}C_{1}\omega^{p_{1}}cos(\pi p_{1}/2)+R_{b}^{2}C_{1}^{2}\omega^{2p_{1}}+1}+\frac{cos(\pi p_{2}/2)}{C_{2}\omega^{p_{2}}}$$

(11) $$Z_{i}=\frac{R_{b}^{2}C_{1}\omega^{p_{1}}sin(\pi p_{1}/2)}{2R_{b}C_{1}\omega^{p_{1}}cos(\pi p_{1}/2)+R_{p}^{2}C_{1}^{2}\omega^{2p_{1}}+1}+\frac{sin(\pi p_{2}/2)}{C_{2}\omega^{p_{2}}}$$

Here, *C*_1_ is the capacitance of CPE1 at the bulk of the electrolyte; *C*_2_ is the CPE2 capacitance at the electrode-electrolyte interface; *p*_2_ is the offset from the real axis and *p*_1_ is the offset of the semicircle from the imaginary axis. The fitting parameters in the EEC are listed in Table 1. As seen in Table 1, *C*_1_ and *C*_2_ increased with increasing salt concentration as the number density of ions increases and they transport from the bulk of the electrolyte to the surface of the electrodes. In addition, the conductivity is also increased with increasing salt amount due to the dissociation of more salts to ions and the decrease in the *R_b_* value as seen in Figure 2a–d. 

The electrode polarization is responsible for appearing the spike in Figure 2a–d at the interfaces between electrodes and electrolytes owing to blockage of ions at the electrode-electrolyte interfaces. Consequently, the electrode polarization outcome is caused by the formation of an electric double layer, resulting in free charge accumulation at the interfaces between electrodes and electrolytes. The linear increase in impedance in low frequency region in Figure 2 is expected to be a straight line (90 degree) parallel to the imaginary axis. However, there is an inclination by nearly 45° from the straight line due to the electrode polarization which causes to block of ions at the surface of the electrodes as seen in Figure 2a–d. Notably, the semicircular feature in the high frequency region has significantly diminished as KI was increased to 30 wt % and 40 wt %.

Equation (1) is used to compute the dc ionic conductivity by measuring the sample thickness and *R_b_* and the conductivity values are summarized in Table 2. As seen in Table 2, the dc conductivity increased when concentration of salt increased as more ions formed at higher salt concentration. From Equation (1), the lowest *R_b_* value shows the highest ionic conductivity [28]. It can be noted that the bulk resistance decreases with increasing the KI salt concentrations from 10 to 40 wt %. *µ* is related to the number density (*n*) and electrolyte conductivity (*σ_dc_*) by Equation (12) [29]

(12) $$\sigma_{dc}=ne\mu$$

where, *n* is the density of the charge carrier, *µ* denotes mobility of ions, and *e* denotes an electronic charge. It was established that the polymer electrolytes must have a dc ionic conductivity in the range between 10^−3^ and 10^−5^ S cm^−1^ in order for it to be used in electrochemical devices [25,30,31]. Researchers have discovered that the conductivity value in this range is desirable for use in energy devices [25,30,31]. Shuhaimi et al. [32] were obtained the highest conductivity of 2.1 × 10^−6^ S cm^−1^ for the system of MC-NH_4_NO_3_ based biopolymer electrolyte.

As all the impedance data composed of a semicircular feature and a linear impedance, transport parameters including *D*, *μ* and *n* of ions are determined using the following equations [26,28]. The *D* of the ions is calculated using Equation (13),

(13) $$D=\frac{{\left(K_{2}\varepsilon_{o}\varepsilon_{r}A\right)}^{2}}{\tau_{2}}$$

where *ε_r_* is the dielectric constant, *τ_2_* is the reciprocal of angular frequency, which corresponds to the lowest value of *Z_i_*.

The *µ* of the ions is determined using Equation (14)

(14) $$\mu =\left[\frac{eD}{K_{B}T}\right]$$

where *T* is the absolute temperature and *K_b_* is the Boltzmann constant.

Since the *σ_d__c_* is given by Equation (12), the number density of ions (*n*) is calculated using Equation (15)

(15) $$n=\left[\frac{\sigma_{dc}K_{b}T\tau_{2}}{{\left(eK_{2}\varepsilon_{o}\varepsilon_{r}A\right)}^{2}}\right]$$

Table 2 lists the ion transport parameters for each electrolyte system.

Based on Table 2, the *D* increased as the KI concentration increased from 10 to 40 wt %. The identical tendency is seen by *μ* as listed in Table 2 where *μ* increased. The increase of *μ* and *D* is related to the increase of chain flexibility with the existence of slat [28]. Consequently, an improvement of conductivity is resulted.

**Figure 3:**
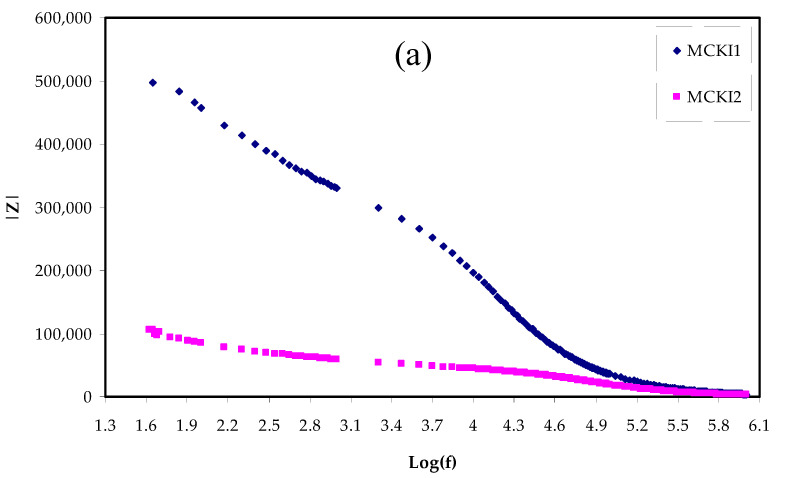

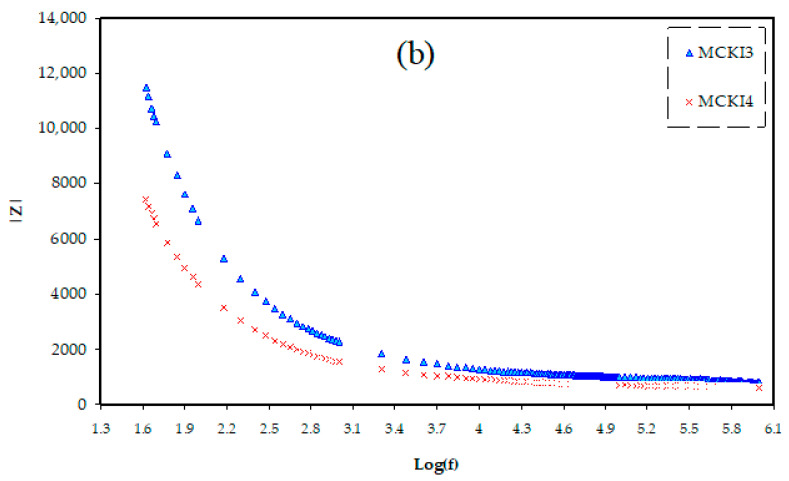
Bode plots for (**a**) MCKI1 and MCKI2 and (**b**) MCKI3 and MCKI4 electrolyte samples.

Figure 3a,b show the Bode plot for each electrolyte film at room temperature. An earlier study [33] indicated that the capacitive region is a plateau region between 10^−2^ Hz and 100 Hz. However, this feature is not observed in Figure 3 because of the limitation of frequency of our measuring equipment. As described at the EIS plots, the semicircle is associated with ion transfer in the electrolyte and the linear feature arises from ions diffusion and therefore their accumulation at the interfaces between electrode and electrolyte [33] which leads to an electrical double-layer capacitances. It was shown that, by increasing the amount of salt from 10 wt % to 40 wt %, the linear feature increased and the resistance reduced from 3.3 × 10^5^ to 8 × 10^2^ Ω, because of the more carrier density. As seen in Figure 3a the electrolyte film has high charge transfer resistance (*R_ct_*) while with increasing salt the *R_ct_* decreased as shown in Figure 3b. The dispersion region between 40 Hz and 40,000 Hz is ascribed to the phenomena of ion diffusion and the high-frequency region is ascribed to the *R_ct_*. In Figure 2 and Figure 3, it is seen that the sample loaded with 40 wt % of KI has the lowest *R_ct_* and hence a large conductivity resulted. Therefore, the Bode plot supports the result measured from the impedance study.

### 3.2. Dielectric Properties

Complex electric modulus, defined as the inverse of complex relative permittivity, can be a significantly powerful tool for analyzing dielectric behavior of a polymeric insulating material, especially at relatively high temperatures, where complex permittivity usually becomes very high due to electrode polarization and carrier transport. The core of electrochemical devices are ions conducting solid electrolytes, and its electrical properties investigation such as *σ_dc_*, ε*, and electric modulus (M*) are essential to understanding the ions transport process [21]. The real part (*ε*′) is related to ion storage efficiency or polarizing ability, while the imaginary part (*ε*″) is the necessary energy for dipole alignment [34]. The *ε*′ and *ε*′′ are determined using Equations (2) and (3).

**Figure 4:**
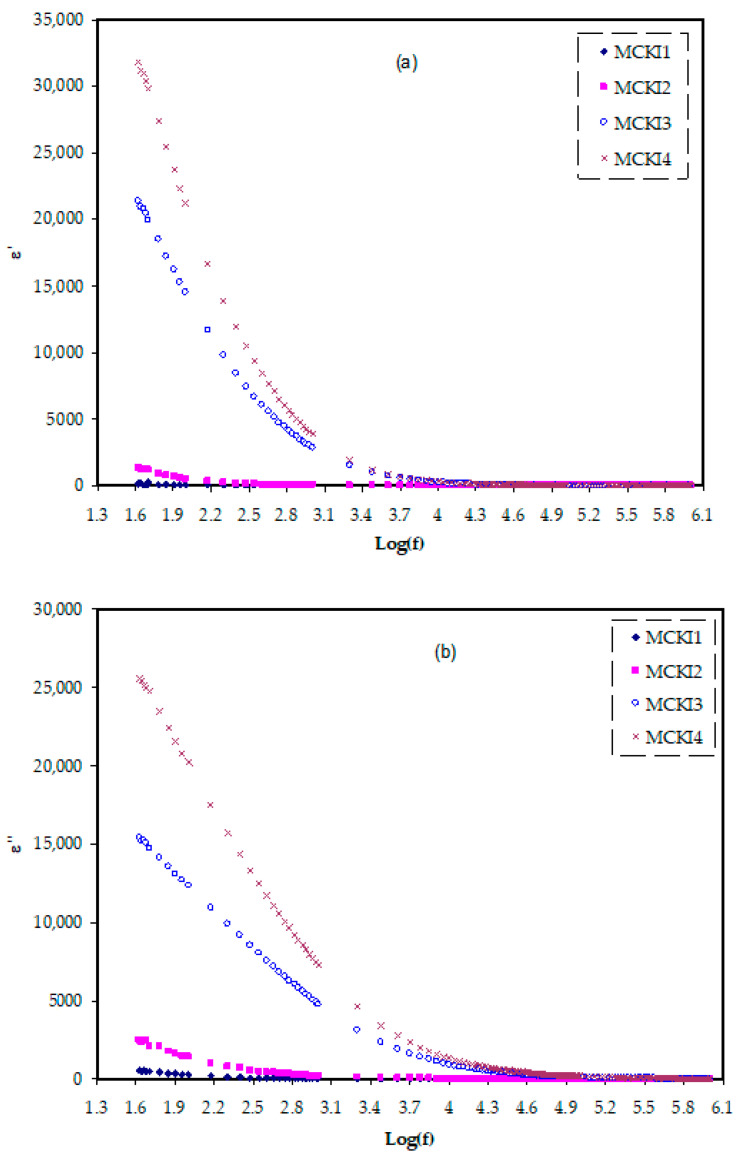
Dielectric plot for (**a**) *ε*′ and (**b**) *ε*″ variation against frequency for the MCKI samples.

Figure 4a,b display the frequency dependency of the *ε*′ and *ε*″ for the MC polymer incorporated with various concentrations of KI salt. It can be noted that the system integrated with 40 wt % of KI has the highest dielectric constant at a low-frequency region. It might be owing to the electrode polarization and also space charge effects. The rise in dielectric constant can be explained by the high charge carrier concentration of the system and its amorphous composition [35]. It is seen that as the salt content (KI) increases, the *ε*′ and *ε*″ increase. This is in agreement with the increase in number density and mobility of ions when the KI content increased as shown in Table 2. Both of the *ε*′ and *ε*″ values are elevated at low frequencies and decreased as frequency rises, indicating polarization effect due to charge accumulations near electrodes at low frequency and dipoles do not obey the field variation at a high dispersion frequency region [36]. The dielectric values remain stable at high-frequency regions due to the interfaces of the electrode–electrolyte become marginal as the frequency increases. The decreased value of both *ε*′ and *ε*″ with increasing frequency means that the electrolyte films are non-Debye behavior [37].

The *Z_r_* and *Z_i_* data were achieved from the EIS data and then used to determine the *ε*′ and *ε*″ data. The *ε*′ and *ε*″ were used to find the *tan δ*. The *tan δ* is the ratio between energy disperse and energy stored in a periodical field which is also called dissipation factor [23] and it is determined using Equation (16).

(16) $$tan\;\delta =\frac{\varepsilon \prime \prime }{\varepsilon \prime }$$

**Figure 5:**
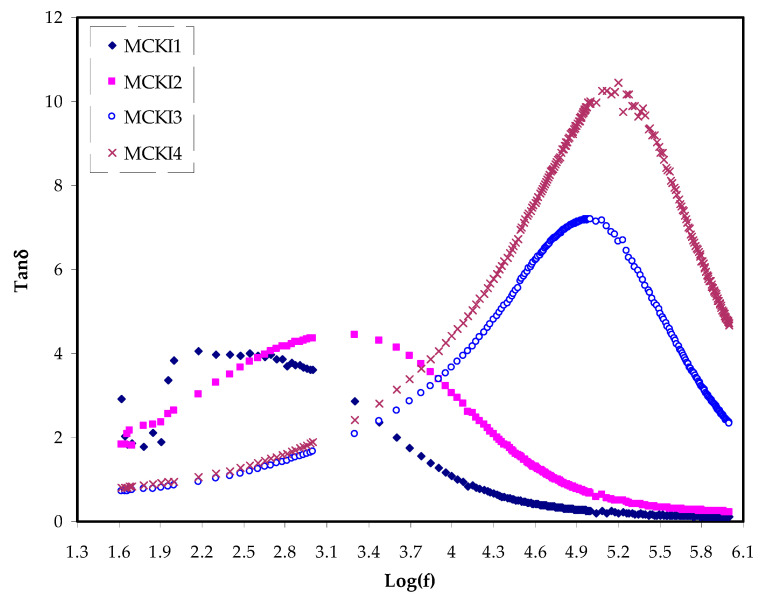
(*tan δ*) spectra versus frequency at room temperature for the MCKI electrolytes.

Dielectric loss is the energy dissipation by the transfer of charges in an alternating electric field as polarization switches direction. When the electric field is applied, polarization happens and charges are moved relative to the electric field. Dielectric loss causes a decrease in the overall electric field. The total amount of polarization that can happen in a dielectric relies on the molecular symmetry of the insulator material and is known as dipole moment. The influence of the dipole moment in a dielectric material is called loss tangent. The ratio of *ε*″ to *ε*′ is defined as *tan δ*, where *δ* denotes a loss angle. The *tan δ* is determined using the relation below [23]. Loss tangent (*tan δ*) was further investigated for the MC polymer incorporated with various KI concentrations. Figure 5 shows the loss tangent (*tan δ*) spectra versus frequency at room temperature. The relation between loss tangent and frequency reveals some interesting behavior. Overall, the loss tangent increases with increasing the applied frequency due to the domination of the Ohmic components. It reaches a high value at a certain frequency, and followed by decreases at a high frequency, owing to the increasing nature of the reactive components [38]. Notably, MCKI4 displays the highest shift to the high frequency and the maximum value relative to the other samples due to the value of dielectric constant *ε*′ for the MCKI4 as shown in Figure 4a [39]. The presence of the peaks at a characteristic frequency can be argued for indicating the presence of dipole relaxation in the electrolytes. It has been reported that improving the segmental motion of polymer chains decreases the relaxation time, allowing the transport process easier. This is expressed mathematically as $\tau =1/2\pi f_{max}$, where *τ* is the ionic charge carrier’s relaxation time [40].

**Figure 6:**
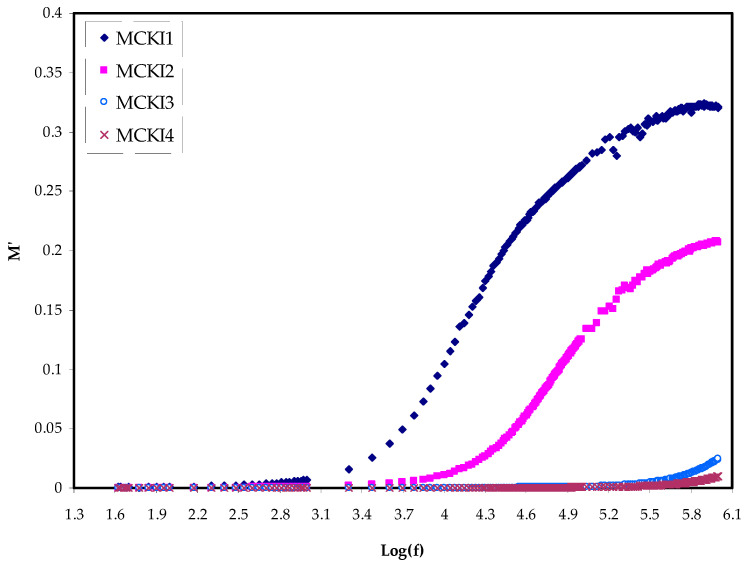
Electric modulus plot of *M_r_* against log(f)for the MCKI samples.

**Figure 7:**
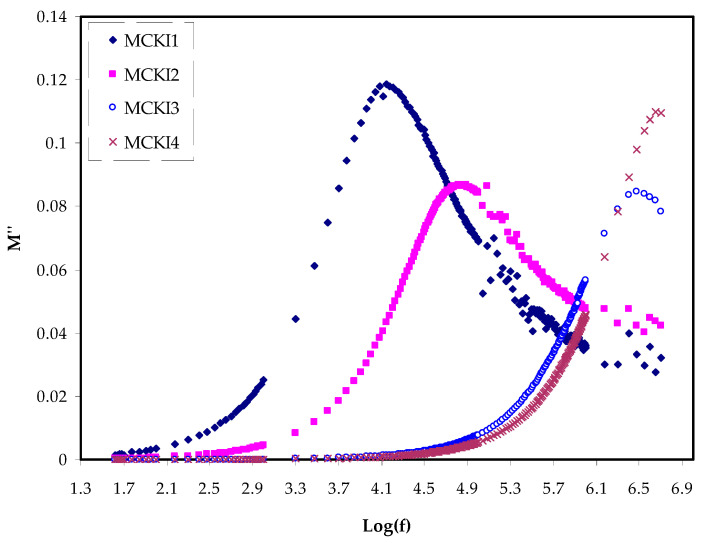
Electric modulus plot of *M_i_* against log(f) for the MCKI samples.

The real, *M_r_* and imaginary, *M_i_* components of the electric modulus M* against frequency for the MCKI based solid polymer electrolytes are shown in Figure 6 and Figure 7, respectively. The *M′* and *M″* are determined using Equations (4) and (5).

From the figures, *M_r_* values are noted to decrease with decreasing frequencies until they reach zero, meaning that the polarization was eliminated. Therefore, the *M_r_* values rise with increasing frequency and at the highest frequency, the maximum *M_r_* was obtained. This could be attributed to the fact that the relaxation process occurs at various frequency values [41]. The observed dispersion is essentially as of conductivity relaxation covering several frequencies, indicating the presence of *τ* that has to occur with a loss peak in the figure of the imaginary part of the dielectric modulus versus frequency. As *M_i_* has clearly a lower value at a low frequency, this may be attributed to the higher capacitance coupled with the polarization effect. No peak is present in Figure 6 along with its entire frequency range. It could be referring to the *M_r_* which is equivalent to the *ε*′ in the ε* representation, which *M_r_* shows the material’s potential for energy conversion [42].

### 3.3. FTIR Study

**Figure 8:**
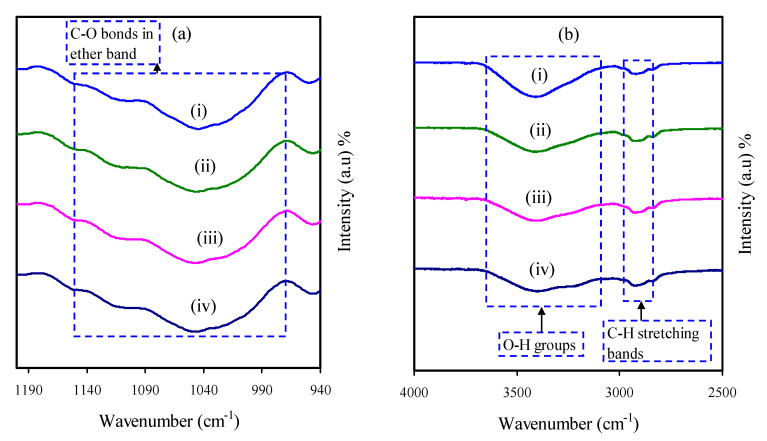
FTIR spectra of the MCKI samples at a wavenumber of (**a**) 940–1200 cm^−1^ and (**b**) 2500–4000 cm^−1^ for (i) MCKI1 (ii) MCKI2, (iii) MCKI3, and (iv) MCKI4 electrolyte samples.

The technique of FTIR spectroscopy has been used to investigate the interactions between ions and atoms of the MCKI electrolytes. Also, such interactions can lead to the changes in the vibration modes of the polymer electrolyte. The FTIR spectra of the pure MC and MCKI based solid polymer electrolyte over the wavenumber range of 940–4000 cm^−1^ are displayed in Figure 8a,b. The broad peak observed at around 1050 cm^−1^ corresponds to the antisymmetric stretch of an asymmetric oxygen bridge in its cyclohexane ring of pure MC. The water contamination from the KI salt causes a broad peak at 3400 cm^−1^ of the O-H stretching band. The observed peak intensity changes as the weight percent of KI salt was increased from 0 to 40 % in the MC-KI electrolyte systems, as shown in Figure 8a,b [43,44]. A peak that appears in the wavenumber region of 2800–2950 cm^−1^ is corresponding to the C-H stretching mode of methylcellulose. Through the inclusion of KI salt, the peak seems to shift slightly from 2850 cm^−1^ to 2990 cm^−1^. This shift of the peak may be an indication of the complexation of K^+^ cation and the MC host polymer. However, the slight change in the C-O ether bands indicates that the complexation did not considerably modify the molecular structure of the MC host polymer. Furthermore, the change in peak intensity with increasing KI concentrations supports that the presence of KI salt in the system has a significant impact on the conductivity of the MCKI electrolyte systems [45].

### 3.4. EDLC Study

#### 3.4.1. Study of the TNM

**Figure 9:**
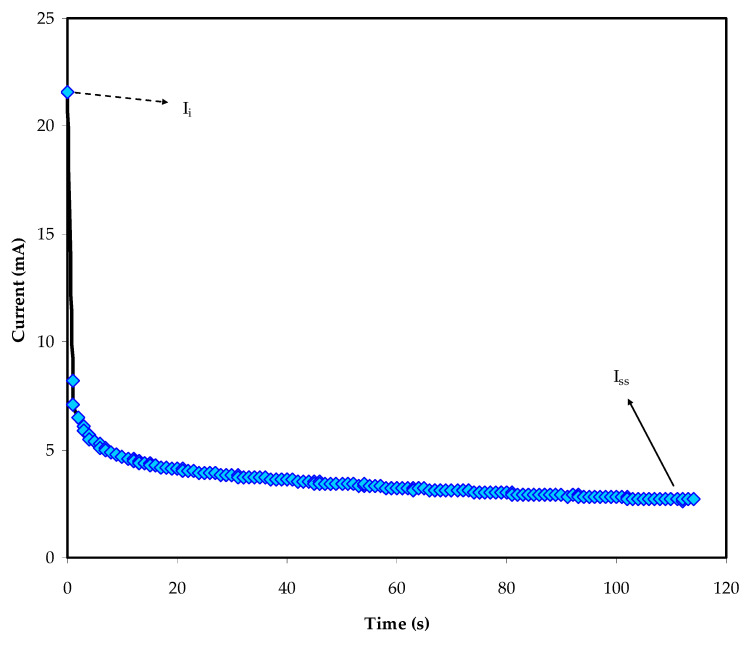
DC polarization curve of current versus time for the MCKI4 sample.

Both ions and electrons in polymer electrolytes are generally responsible for their conductivity. Through this technique, the dominant charge carrier in the polymer electrolyte can be evaluated [46]. Figure 9 shows the current versus time plot, obtained by dc polarization at 0.2 V, for the MCK1_4_ film. Equations (6) and (7) were used to determine the *t_ion_* and *t_el_* of the MCKI4 film.

According to Figure 9, the initial total current was found to be 22 µA [47]. Therefore, a large drop is observed over time until being constant in a completely depleted case due to the transport of ionic species from the bulk of the MCKI4 electrolyte to the electrode-electrolyte interfaces. When the cell reaches the steady state, it is polarized, and the residual current is only carried by electrons due to the stainless-steel electrodes block both cations and anions while allowing only electrons to move through it. In this analysis, the measured *t_el_* value was 0.12 and the *t_ion_* was found to be 0.88, which is close to an ideal value of 1 [28], indicating that ions in the MCKI4 film is the majority charge carrier [48]. The finding obtained in this work is comparable with the *t_ion_* value of 0.86 as reported by Aziz et al. for the polymer electrolyte system of chitosan: dextran: NH_4_Br [49].

#### 3.4.2. LSV Study

**Figure 10:**
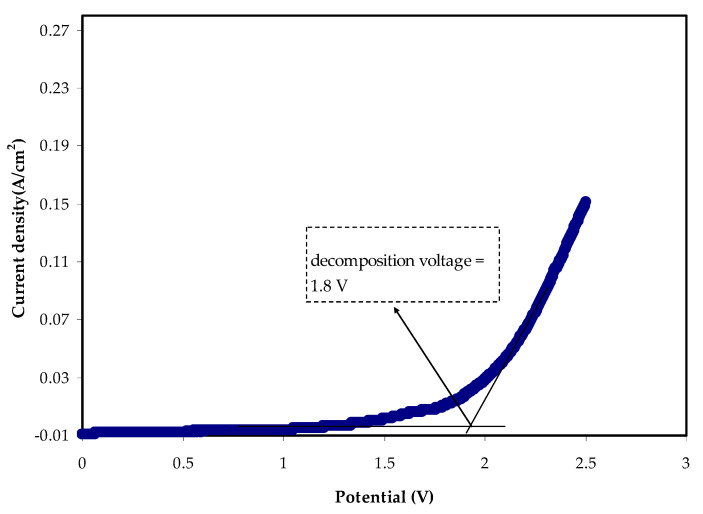
Current versus potential for the highest conducting (MCKI4) sample.

The potential stability of the polymer electrolyte systems needs to be established for energy device research. The absolute potential limit of the electrolytes can be computed in terms of linear sweep voltammetry LSV examination [50]. The LSV for the most conducting sample MCKI4 at 10 mV/s is shown in Figure 10, in which the potential was scanned from 0 to 2.5 V. When potential approaches to 1.8 V, the electrolyte reaches decomposition voltage as revealed by a significant increase in current values. Also, there is no evidence of a redox reaction occurring within the potential window until 1.8 V. Based on the previous study, the electrolyte with the potential window of 1.8 V is sufficient to be used for application in proton energy devices [51]. Other research findings relating to MC-based biopolymer electrolytes are comparable to this work. According to Kadir et al. [52], MC-based electrolytes displayed a decomposition voltage of 1.53 V when NH_4_Br and glycerol were used as the ionic source and plasticizer, respectively. The breakdown potential of 1.9 V was reported for the biopolymeric system of starch-chitosan-NH_4_I with the existence of glycerol [53], which is similar to this study.

#### 3.4.3. Cyclic Voltammetry (CV) Study

**Figure 11:**
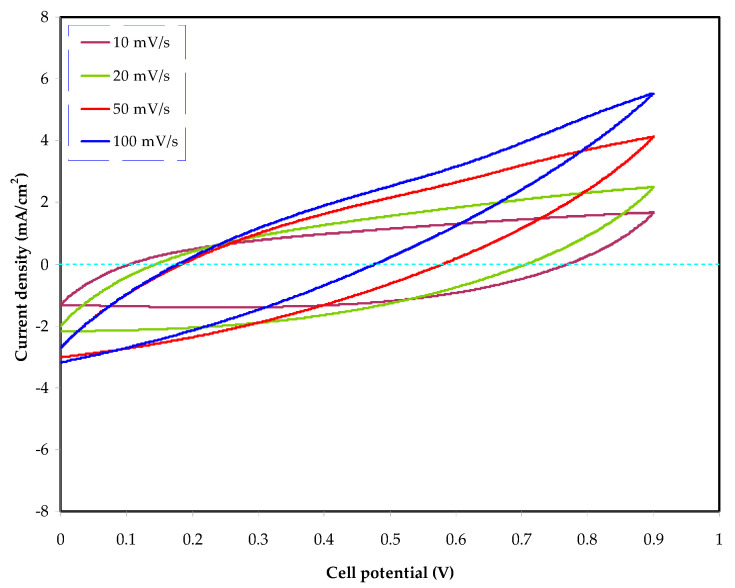
Cyclic voltammograms for the assemble EDLC in the potential range of 0 to 0.9 V.

CV as an insightful technique can be employed to examine the EDLCs in terms of both qualitative and quantitative features [54]. It is used to further evaluate the efficiency of the MCKI4 electrolyte in the construction of the EDLC. The CV responses of the MCKI4 electrolyte at various scan rates of 10, 20, 50, and 100 mV/s are shown in Figure 11 in the potential range of 0 to 0.9 V.

The CV response has a rectangular form, indicating that the current is independent of the potential. However, the shape of the cyclic voltammogram (CV) deviates from the rectangular shape when the scan rate increases [55]. The CV in Figure 11 showed that the EDLC exhibits a capacitive behavior, indicating that the system of the energy storage is a non-Faradaic mechanism. In this process, the charge stored in the EDLC system comes from ion accumulation at the electrode/electrolyte interfaces. As a consequence, ion accumulation and adsorption occur in the place of deintercalation and intercalation via a non-Faradaic mechanism. In addition, ions from the bulk of the electrolyte form a charge double-layer, which then saves potential energy [56,57]. Notably, the CV displays a leaf-like shape with no redox peaks. The CV profile revealed a little divergence from its rectangular form at higher scan rates, which may be due to the porosity of the electrodes as well as internal resistance. The porosity of the carbon electrodes induces a relatively high internal resistance, which causes the CV to appear leaf-like in shape [58]. Since the CV possesses no redox peaks, it is reasonable to infer that a quick Faradaic reversible reaction has not occurred [59].

**Figure 12:**
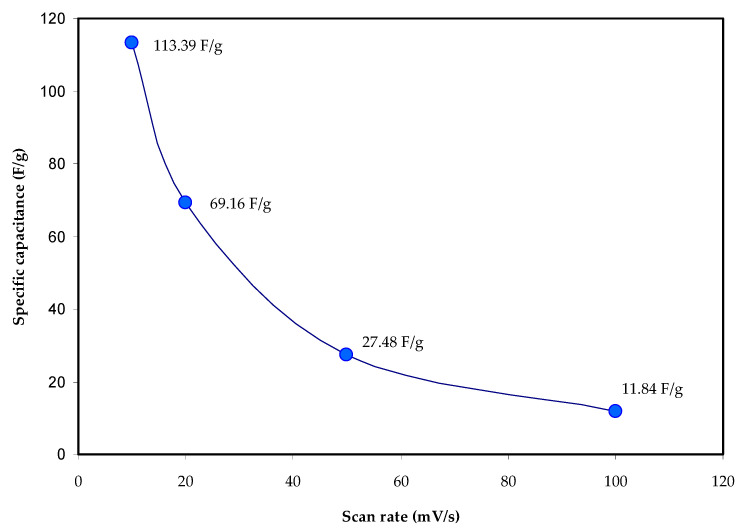
The calculated specific capacitance, *C_s_* for the assembled EDLC at a different scan rate.

The specific capacitance (*C_s_*)are determined using Equation (8) by measuring the area of the CV profile, mass of the activated carbon electrode, scan rate, and the initial and final values of applied voltage. The measured specific capacitance values, *C_s_* using CV curves for the assembled EDLC at different scan rates are shown in Table 3 and Figure 12. The calculated *C_s_* value of 113.39 F/g at the sweep rate of 10 mV/s decreased to 11.84 F/g at 100 mV/s. The low *C_s_* value at high scan rates is attributed to the high energy loss caused by the decrease in the density of stored charges, which results in a lower *C_s_* value [60]. Table 4 displays the measured *C_s_* value of the EDLC for several systems based on solid biopolymer electrolytes mentioned in the literature. Interestingly, the *C_s_* value obtained in this work is high and comparable to some of these results.

## 4. Conclusions

In conclusion, a biopolymer-based electrolyte using methylcellulose (MC) incorporated with various content of potassium iodide (KI) salt is crucial for EDLC device applications. The EIS outcome shows that the resistance of the transfer of charge at the bulk of the electrolyte reduced from 3.3 × 10^5^ Ω to 8 × 10^2^ Ω with KI concentration increased from 10 wt % to 40 wt % due to an increase in the charge carrier density. The highest conductivity of 1.93 × 10^−5^ S/cm was obtained for the electrolyte doped with 40 wt % of KI. The dielectric analysis further verified the conductivity trends. The results from the FTIR spectra indicated that the complexation between (K^+^) cation and (MC) host polymer has occurred through intensity variations of bands. TNM measurements stated that the ions were the dominant charge carrier, as the (*t_ion_*) was identified to be 0.88. LSV analysis showed that the most conducting sample has an electrochemical stability window up to 1.8 V, verifying the suitability of the electrolyte for EDLC application. The CV response displayed its capacitance behavior, where no visible redox peak has appeared. A relatively high value of the specific capacitance *C_s_* (113.39 F/g) was obtained at the scan rate of 10 mV/s.

## Figures and Tables

**Table 1:** The EEC fitting parameters for the systems fabricated.

Sample	P_1_ (rad)	P_2_ (rad)	C_1_ (F)	C_2_ (F)
MCKI1	0.90	0.41	2 × 10^−10^	3.33 × 10^−7^
MCKI2	0.87	0.42	4 × 10^−10^	1.43 × 10^−6^
MCKI3	0.76	0.65	6.67 × 10^−9^	2.44 × 10^−6^
MCKI4	0.73	0.62	1.11 × 10^−8^	4.55 × 10^−6^

**Table 2:** Numerical values of *σ_dc_***,** *D*, *µ*, and *n* at ambient temperature.

Sample	*σ_dc_* (S cm^−1^)	*D* (cm^2^ s^−1^)	*µ* (cm^2^ V^−1^ s)	*n* (cm^−3^)
MCKI1	4.65 × 10^−8^	1.15 × 10^−9^	4.48 × 10^−8^	6.49 × 10^18^
MCKI2	3.59 × 10^−7^	1.35 × 10^−9^	5.26 × 10^−8^	4.25 × 10^19^
MCKI3	1.35 × 10^−5^	2.00 × 10^−9^	7.78 × 10^−8^	1.08 × 10^21^
MCKI4	1.93 × 10^−5^	2.13 × 10^−9^	8.29 × 10^−8^	1.45 × 10^21^

**Table 3:** Specific capacitance (*C_s_*) of the EDLCs using CV curves.

Scan Rates (mV/s)	Specific Capacitance, *C_s_* F/g
10	113.39
20	69.16
50	27.48
100	11.84

**Table 4:** Specific capacitance (*C_s_*) of the EDLCs using different polymer electrolytes at room temperature.

Biopolymer Electrolytes	Specific Capacitance, *C_s_* F/g	Scan Rates (mV/s)	Reference
Chitosan-PVA-Mg(CF_3_SO_3_)_2_:glycerol	32.69	10	[3]
Starch-LiClO_4_	8.7	10	[7]
MC-NH_4_NO_3_-PEG	38	1	[45]
MC-chitosan-NH_4_SCN	66.3	10	[33]
Carboxymethyl cellulose-NH_4_NO_3_	1.8	Not stated	[61]
MC-chitosan-NH_4_I-glycerol	9.97	10	[62]
Cellulose acetate-LiClO_4_	90	10	[63]
Chitosan-NH_4_Br-glycerol	7.5	10	[64]
MC-Starch-LiClO_4_-glycerol	45.8	10	[65]
MC-KI	113.39	10	This work

## Data Availability

The data presented in this study are available on request from the corresponding author.
